# Sialadenitis as chickenpox complication in a 6-year-old girl

**DOI:** 10.1016/j.idcr.2021.e01052

**Published:** 2021-01-20

**Authors:** A. Betkiewicz, M. Fornalczyk, K. Urban, B. Jarecka, A. Obuchowicz

**Affiliations:** a Department of Paediatrics, Specialist Hospital No 2 in Bytom, Poland; b Department of Paediatrics in Bytom, School of Health Sciences in Katowice, Medical University of Silesia in Katowice, Poland

**Keywords:** Chickenpox, Complications, Sialadenitis, Child

## Abstract

Chickenpox is considered as a mild disease, but sometimes it is associated with complications. Among them sialadenitis is mentioned sporadically. We describe a case of the 6-year-old girl suffering from complicated chickenpox. On the basis of clinical data and ultrasound image we diagnosed in her inflammation of both submandibular glands. Moreover, the ultrasound image suggested possibility of an inflammation developing in the left parotid gland.

## Highlights

• In the 6.year old girl with chickenpox we diagnosed an typical complication in the shape of sialadenitis (especially submaxillitis).
• Within the last twenty years, only in one child the relationship between sialadenitis and varicella-zoster virus infection has been suggested.
• Our observation has important clinical significance.

## Introduction

Chickenpox is generally considered a mild disease. Although this is most often the case, it is sometimes associated with complications. The most common are secondary bacterial infections, usually developing on the basis of a damaged follicular eruption. These include bacterial infections with multiple locations, most often caused by Group A *Streptococcus* and *Staphylococcus* bacteria. There may also be organ complications (including neurological, nephrological and ophthalmological ones) related directly to Varicella zoster virus (VZV) infection or an autoimmune reaction [1,2]. The following very rare conditions have also been mentioned in case reports: multi-organ failure [3], aplastic anaemia [4], gastritis [5].

According to the Polish Institute of Hygiene, the number of chickenpox cases in Poland within the last ten years ranged from 149 to 221 thousand per year. In particular years, 0.55−0.76% of patients infected with VZV required hospitalisation [6]. In the years 2010–2019, 66 children with severe chickenpox and/or concomitant diseases were hospitalized at the Department of Paediatrics. Among them was a girl whose case study is presented here.

The aim of the study is to point out to the possibility of occurrence of sialadenitis as chickenpox complication.

## Case study

A 6-year-old girl, previously healthy, suffering from chickenpox for 5 days, was admitted to the department in a serious condition due to substantial, painful swelling in the submandibular area and lower 1/2 of the left cheek that developed in the course of the illness. The girl's condition worsened at home after 2 days of mild course of chickenpox. Oral administration of acyclovir (4 × 400 mg was began). None the less there occurred drowsiness, fever up to 39.8 °C, vomiting and hard swelling of the above-mentioned area, increasing in size. Within its boundaries, a pronounced erythema (2 × 2 cm) was observed in the place of two chickenpox eruptions scratched by the child. On admission to the hospital, the girl was sleepy, dehydrated, feverish up to 38 degrees Celsius, with minimal ability to open her mouth. Most of the smallpox eruptions were in the healing phase. New ones were appeared for 2 days (on the day of admission and on the next day – jointly it was 6 days of new lesion formation). Apart from the very painful lesion described above, also the skin on the trunk and upper limbs was highly erythematous. Laboratory tests showed high concentration of CRP (80.49 mg/L on day 1, 143.31 mg/L on day 2) (normal value < 5 mg/L) and procalcitonin (26.2 ng/mL on day 1, 100.0 ng/mL on day 2) (normal value < 0,5 ng/mL). Blood culture was negative. Taking into account the clinical features of soft tissue inflammation in the area of ​​the left side of the mandibular body and the level of the inflammatory markers, ceftriaxone (i.v.) and vancomycin (i.v.) were introduced in addition to the ongoing administration of acyclovir (to 5 days from the begining of this therapy). Abdominal ultrasound examination showed only moderate enlargement of the left lobe of the liver. Ultrasound image of the neck and salivary glands showed inflammation features of the left submandibular gland. It was swollen, with an inhomogeneous echo, significantly enlarged, at least 32.5 × 24 × 32.5 mm in size (approximate measurement due to blurred contours). Moreover, enlarged upper and middle cervical lymph nodes with reactive morphology up to 9 mm in the short axis, more numerous on the left side were also visible. The changes were progressing during the first two days of hospitalisation. Both submandibular glands were enlarged in palpation and usg image (at least 40.7 × 21 × 26 mm in size - indicative measurement), swollen, with inhomogeneous echo. The swelling of the subcutaneous tissue above the left part of mandibular body and bilaterally enlarged cervical lymph nodes were observed. The left parotid gland was not enlarged, but in repeated studies it showed a slightly inhomogeneous echo and the presence of several enlarged reactive lymph nodes within it. After 4 days of septic fever, the body temperature normalized and the patient`s general condition improved. The size and soreness of the inflammatory infiltration in the subcutaneous tissue started decreasing from the 7th day while the ultrasound image suggested the formation of an abscess above the left part of mandibular body. The ultrasound image of the right submandibular gland normalized after 5 days, and after a week of hospitalization the left submandibular gland was only moderately enlarged (20 × 14.5 × 19 mm) and had a blurred structure. A surgeon incised the abscess and the culture of the abscess contents revealed the presence of *Streptococcus pyogenes* which was sensitive to antibiotics administered to our patient. The patient was discharged home after 14 days of treatment, in good condition and with normal laboratory test results.

## Discussion

As emphasized in literature, regardless of the country of occurrence, chickenpox - despite its mostly mild course - can cause serious complications [2,1,7] To some extent, the type of complications is age-dependent. Infectious symptoms dominate in younger children, up to 4 years of age. The most severe ones are caused by *Streptococcus pyogenes and Staphylococcus aureus* and affect the skin and subcutaneous tissue, fascia, respiratory system, bones and joints [2,8]. In the most severe cases, the patient may die [1]. Also in our patient bacterial infection was probably the main reason of health worsening and significant increase of inflammatory markers. Additionally, neurological complications are observed in older children as a consequence of immune reaction [2]. Apart from encephalitis or cerebellitis, the occurrence of cerebral ischemia and infarction resulting from cerebral vasculitis was described in children, occurring 3–4 months after varicella [9,10]. Despite the extensive literature on complications of chickenpox, sialadenitis has been reported sporadically. A 15-year-old boy suffering from dermatomyositis and diabetes mellitus, in whom submandibular gland oedema persisted for a year after suffering from chickenpox, was described. Radiological examination demonstrated calcifications within the gland. Based on the possibility of the formation of concrements in the lung tissue related to the infection with *Herpes* virus, the authors believed that VZV infection could have contributed to the formation of these calcifications [11]. An 8-year-old girl with cervical shingles, paralysis of the facial nerve and oedema of the right submandibular region was also described. However, not only IgM antibodies against VZV, but also against mumps virus were found in the child [12]. If the ultrasound image in our patient had only shown inflammation of the left submandibular gland, it could have been be assumed that it was an inflammatory reaction related to a bacterial infection in close anatomical area. However, the image also demonstrated the inflammation of the second submandibular gland and suggested the possibility of inflammation developing in the left parotid gland. A significant reduction in the size of the left submandibular gland was found by ultrasound after 7 days, despite the formation of the abscess in the subcutaneous tissue. This course seems to indicate inflammation of the salivary glands caused by VZV. We don’t know to what extent it worsened the general condition of the girl. The possibility of the presence of VZV in the salivary glands was confirmed by the study revealing Varicella zoster virus DNA in patients with Ramsay Hunt syndrome. The authors confirmed the presence of DNA in 72 % of salivary samples of the submandibular glands and 57 % of the parotid glands, using the PCR method [13]. The possibility of developing shingles after childhood chickenpox, due to VZV latency in sensory ganglia is a serious health risk associated with numerous complications [8,14,10] Vaccination is the most effective method of preventing VZV infection. After the introduction of the two-dose vaccination regimen in the USA (2007), the number of cases, hospitalizations and deaths decreased by over 90 % [8,15]. In Poland, since 2020, vaccination has been obligatory for children up to 12 years of age, at risk of infection due to clinical or environmental reasons. The described girl did not meet the above criteria and was not vaccinated. Also she wasn’t immunocompromised and nothing suggested a possibility of severe course of the disease.

## Conclusions

1 The possible complications of VZV infection to be considered include sialadenitis.
2 The possibility of a complicated course of chickenpox indicates the need to inform parents about such a risk and convince them to inoculate their children against this seemingly “harmless” disease.

## Author statement

All authors accept the text with changes made in response to reviewers` comments.

## Funding

The research did not receive any specific grant from funding agencies in the public, commercial, or not-for-profit sectors.

## Ethical approval

This study didn’t require ethics committee approval.

## Consent

Written informed consent was obtained from the patient`s mother for publication of this case report.

## CRediT authorship contribution statement

**A. Betkiewicz:** Conceptualization, Writing - original draft. **M. Fornalczyk:** Conceptualization, Writing - original draft. **K. Urban:** Investigation. **B. Jarecka:** Visualization, Writing - review & editing. **A. Obuchowicz:** Supervision.

## Declaration of Competing Interest

The authors report no declarations of interest.
